# A Reweighted *ℓ_1_*-Minimization Based Compressed Sensing for the Spectral Estimation of Heart Rate Variability Using the Unevenly Sampled Data

**DOI:** 10.1371/journal.pone.0099098

**Published:** 2014-06-12

**Authors:** Szi-Wen Chen, Shih-Chieh Chao

**Affiliations:** 1 Department of Electronic Engineering, Chang Gung University, Tao-Yuan, Taiwan; 2 Heathy Aging Research Center (HARC), Chang Gung University, Tao-Yuan, Taiwan; University of Navarra, Spain

## Abstract

In this paper, a reweighted *ℓ_1_*-minimization based Compressed Sensing (CS) algorithm incorporating the Integral Pulse Frequency Modulation (IPFM) model for spectral estimation of HRV is introduced. Knowing as a novel sensing/sampling paradigm, the theory of CS asserts certain signals that are considered sparse or compressible can be possibly reconstructed from substantially fewer measurements than those required by traditional methods. Our study aims to employ a novel reweighted *ℓ_1_*-minimization CS method for deriving the spectrum of the modulating signal of IPFM model from incomplete RR measurements for HRV assessments. To evaluate the performance of HRV spectral estimation, a quantitative measure, referred to as the Percent Error Power (PEP) that measures the percentage of difference between the true spectrum and the spectrum derived from the incomplete RR dataset, was used. We studied the performance of spectral reconstruction from incomplete simulated and real HRV signals by experimentally truncating a number of RR data accordingly in the top portion, in the bottom portion, and in a random order from the original RR column vector. As a result, for up to 20% data truncation/loss the proposed reweighted *ℓ_1_*-minimization CS method produced, on average, 2.34%, 2.27%, and 4.55% PEP in the top, bottom, and random data-truncation cases, respectively, on Autoregressive (AR) model derived simulated HRV signals. Similarly, for up to 20% data loss the proposed method produced 5.15%, 4.33%, and 0.39% PEP in the top, bottom, and random data-truncation cases, respectively, on a real HRV database drawn from PhysioNet. Moreover, results generated by a number of intensive numerical experiments all indicated that the reweighted *ℓ_1_*-minimization CS method always achieved the most accurate and high-fidelity HRV spectral estimates in every aspect, compared with the *ℓ_1_*-minimization based method and Lomb's method used for estimating the spectrum of HRV from unevenly sampled RR data.

## Introduction

It is known that the ruling action of Autonomic Nervous System (ANS) is not static [1]. For example, inspecting the beat-to-beat interval, or the so-called RR interval, time series that are directly derived from the Electrocardiogram (ECG), one may notice there exists a certain degree of variation in the RR data sequence. Such intriguing interbeat variations in Heart Rate (HR) actually result from the ANS control. Therefore, one may speculate that the investigations into the modulation of ANS activity may be sought via the analysis of this interbeat variability, or alternatively referred to as the Heart Rate Variability (HRV) [2], [3]. In fact, the analysis results of HRV can be applied for evaluating the condition of the patient's heart in many aspects of clinical applications. Among all these applications, the beat-to-beat variations are generally quantified simply by processing the interbeat interval sequence on time and/or frequency domain. In frequency-domain analysis, for example, the Power Spectral Density (PSD) of HR or RR signal is usually divided into two main frequency components: the Lower Frequency (LF) band ranging from 0.04 Hz to 0.15 Hz, and the Higher Frequency (HF) one ranging from 0.15 Hz to 0.4 Hz. While the HF power is used to serve as an HRV index that reflects the vagal tone, the LF power is considered to reflect the modulation of both the sympathetic and vagal activities [2], [3]. The LF-to-HF power ratio is thus used to reflect the autonomic or sympatho-vagal balance status [4]–[6].

In addition to direct beat-to-beat interval analysis, one may also obtain the information related to ANS control that is not directly measurable simply using a model-based analysis. In this aspect, the Integral Pulse Frequency Modulation (IPFM) model has been used to generate the discrete beat occurrence times from a continuous-time modulating signal that represents the ANS influences on sinoatrial (SA) node [7]–[9]. In fact, the IPFM model has been widely discussed and applied for the generation and analysis of HRV spectra in a number of previous researches in literature [10]–[16]. Among these works, Mitov, Chen and Zhang proposed two IPFM-based methods for HRV spectral analysis, respectively, that both employed unevenly sampled raw RR data as the input [12], [13]. However, both works did not discuss about how to deal with the cases of data deficiency on RR intervals caused by ectopic beats. In addition, Mateo and Laguna defined a novel heart timing signal based on the IPFM model and estimated HRV spectra via various types of Fourier transform of interpolated heart timing signal [14]. Moreover, Mateo and Laguna [15], Solem *et al* [16] further developed two robust HRV spectral estimation algorithms, respectively, that can detect and recover ectopic beats in heart timing signal.

On the other hand, knowing as a novel sensing/sampling paradigm, the theory of Compressed Sensing (CS) asserts certain signals that are considered sparse or compressible can be possibly reconstructed from substantially fewer measurements than those required by traditional methods [17], [18]. Continued research investigations have addressed important issues across many applications spanning from data compression [19], channel coding [20], data acquisition [21], to biomedical signal and image processing. In biomedical signal/image processing, although CS has been successfully applied to a variety of topics, such as Computed Tomography (CT) reconstruction [22] and Magnetic Resonance Imaging (MRI) [23], there still remains a lack in its applications into HRV or the related analysis.

In fact, we may hypothesize that a spectrum of HRV can be described as a sparse or compressible signal since it is widely accepted that major characteristics of a standard HRV spectrum are determined by the amplitudes of three main frequency components only (two are located at the frequency <0.15 Hz; while one is at that >0.15 Hz), and the remainders can be ignored. Therefore, we may take the advantage of using the CS framework to solve for the problem of HRV spectral estimation from substantially fewer measurements. In this aspect, we have combined the use of *ℓ_1_*-minimization based CS framework and the collaboration of IPFM model for deriving the amplitude spectrum of the modulating signal for HRV assessments in a previous work [24]. The primary purpose of our previous work is to establish a CS-based theoretical procedure that can be used to quantitatively characterize the spectrum of HRV using the unevenly sampled RR data. As a result, it was indicated by our previous study that the CS analysis was capable of robustly capturing the spectral information of the fluctuations associated with RR time series of normal heartbeats, even under the situation of a degree of incompleteness in the RR data caused by ectopic or missing beats. It is also worth noting that such a CS-based spectral estimation was unprecedented in HRV analysis.

This study aims at seeking for an enhancement of performance in spectral estimation of HRV by employing a reweighted *ℓ_1_*-minimization based CS method for compressible spectrum recovery that may outperform *ℓ_1_*-minimization in actual practice. First, it should be noted that for most CS algorithms, their successful reconstruction is based on some probability. That is, one cannot ensure each run of algorithms can necessarily get correct reconstruction. However, one can get the correct reconstruction with large probability if some assumptions are satisfied. In contrast, the probability will be smaller when there is noise, or when the sensing matrix is coherent, or when the number of nonzero entries in the true signal is large, i.e., the signal is not sparse enough…etc. In particular, it is known that the global minimum of the *ℓ_1_*-minimization algorithm is not necessary the true optimal solution if the ideal assumptions stated above are not all satisfied. In this aspect, Candès *et al*. proposed an empirical method which tries to solve this problem by employing reweighted *ℓ_1_*-minimization [25].

Since obtaining the true optimal solution in a more practical situation is desired and crucial in our work, seeking for more appropriate choice of algorithm thus motivates this study. Therefore, in this study we further hypothesized that the reweighted *ℓ_1_*-minimization based CS method may be more suitable for spectral estimation of HRV in more practical situations, such as the presence of noise, the coherent sensing matrix, and/or not very sparse HRV spectrum. The objective of this study is to achieve a substantial improvement and enhancement of performance in spectral estimation of HRV using such a novel method. This paper presents a reweighted *ℓ_1_*-minimization based CS algorithm incorporating the IPFM model for spectral estimation of HRV. In fact, the novel method involves solving a sequence of weighted *ℓ_1_*-minimization problems where the weights used for the next iteration are simply computed from the value of the current solution. Numerical experimental results produced by both Autoregressive (AR) model derived simulated signals and a real HRV database of PhysioNet demonstrated that the proposed method can robustly generate the most accurate and high-fidelity HRV spectral estimates, in comparison to our previous *ℓ_1_*-minimization based CS work and Lomb's method used for estimating spectrum of HRV from unevenly sampled RR data.

## Materials and Methods

### 1.1. Integral Pulse Frequency Modulation (IPFM) Model based Spectral Analysis of HRV

Being able to generate impulses from modulating signals, the IPFM model has been widely used as a model underlying the generation of HRV signals. According to the model, first the modulating signal is integrated. When the integral value reaches to a fixed threshold, an impulse is emitted and the integrator is then reset to zero. The model actually provides a functional description of the mechanism by which the ANS modulates HR [11]. Suppose there are *L* of RR intervals, denoted as *RR_i_* = *t_i_−t_i_*_−1_, where *t_i_* is the occurrence time of the *i*th interval, *i* = 1, …, *L* and *t_0_* = 0. IPFM model suggests a linear relation among *t_i_*, modulating signal *m*(*t*) and an IPFM threshold *TR*:
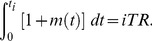
(1)Inspired by the discrete Fourier transform (DFT), we assume
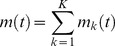
(2)and
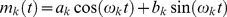
(3)where *ω_k_* = 2*πk*/*T*, *T* is the period of *m_k_*(*t*) while *a_k_* and *b_k_* are real coefficients of cosine wave and sine wave at frequency *ω_k_*, respectively. As a result, (1) becomes
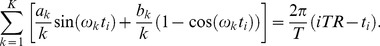
(4)In the context of DFT, the period of *m*(*t*) is equal to the available length of *m*(*t*), *i.e*., *T* = *t_L_*. Thus, when *i* = *L* we can compute
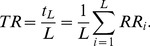
(5)Therefore, *t_i_*, *T* and *TR* can be easily computed, and there are still *L*−1 equations in (4) left, which can be compactly written into the matrix form, **y** = **Ax**, where
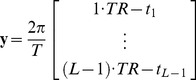
(6)




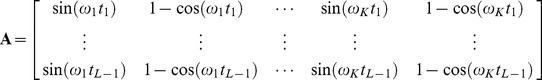
(7)and
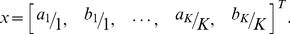
(8)According to the dimension of **A**, the linear systems can be classified as overdetermined, squared and underdetermined, that is,
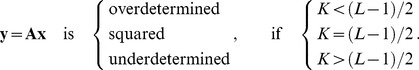
(9)


Obviously, IPFM-based HRV spectra can be estimated by solving the linear system as indicated in (9). Previous studies in literature [12], [13] have developed methods used to estimate IPFM-based HRV spectra in the overdetermined and squared cases, respectively. In this study, we showed that the IPFM-based HRV spectra can be also successfully estimated in the underdetermined case simply by taking advantage of compressed sensing, of which backgrounds and methodology are presented in the subsequent subsections.

### 1.2. Compressed Sensing (CS) Method: by Reweighted ℓ_1_ Minimization

Compressed sensing has been shown to be able to estimate sparse or compressible signals from incomplete measurements [17], [18]. Consider a signal **x** in *R^N^*. **x** is called sparse if most elements of **x** are zero. Similarly, **x** is compressible if most elements of **x** are near zero. Specifically, **x** is *S*-sparse if ||**x**||_0_≤*S*, where *S* is a positive integer and ||·||*_p_* is the *ℓ_p_*-norm operator defined as
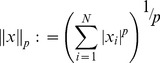
(10)where *p* is non-negative integers. Let us further define the set of *S*-sparse vectors:
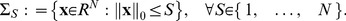
(11)Then, a vector **x′** in *R^N^* can be approximated by a vector ***x*** in Σ*_S_*, and the approximation error of **x′** in *ℓ_p_*-norm, *σ_S_*(**x′**)*_p_*, is
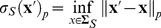
(12)where inf(·) is the infimum operator. If *σ_S_(* **x′** *)_p_* is small enough for *S*, then **x′** is *S*-compressible. Regarding the sampling scheme of CS, unlike the uniform sampling in the Nyquist/Shannon theory, CS employs a linear measurement model by taking a weighted linear combination of samples as
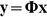
(13)where **y** in *R^M^* is the measurement vector, and **Φ** in *R^M×N^* is the measurement matrix. The interest of CS is that *M*<<*N*, which is considered an underdetermined linear system. This implies that there are infinite solutions. But, on the other hand, one should keep in mind that **x** is sparse or compressible. According to our previous study [24], by taking advantage of the sparsity of **x** we have shown that the spectrum of HRV can be solved in the underdetermined case using the *ℓ_1_*-minimization under the CS framework, where the *ℓ_1_*-minimization is defined as
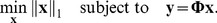
(14)


In this study, a reweighted *ℓ*_1_-minimization [25] is proposed to estimate HRV spectrum since it may require substantially fewer measurements or samples for the task of spectral reconstruction, in comparison to the conventional *ℓ*_1_-minimization, so the CS technique may be more capable of combating the cases of data loss caused by ectopic or missing beats in HRV spectral estimation. In general, reweighted *ℓ_1_*-minimization is a simple iterative algorithm that alternates between estimating **x** and redefining the weights **W**. The iterative algorithm constructs the weights that are proportional to the reciprocal of the magnitudes of elements of **x** obtained from previous iteration in order to allow for successively better estimation of small but nonzero elements of **x**. This improved signal estimation is sufficient to allow perfect spectral reconstruction from even fewer measurements. The complete reweighted *ℓ*_1_-minimization algorithm is described as follows:

1. Set iteration count *k* to zero and *w_i_* ^(0)^ = 1, *i* = 1, …, *N*.
2. Solve the weighted *ℓ*_1_-minimization problem, where
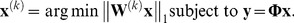
(15)
3. Update the weights: for each *i* = 1, …, *N*,
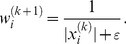
(16)
4. Stop when the solution is converged or when *k* exceeds an allowed maximum number of iterations, denoted as *k*_max_; otherwise, set *k*←*k*+1, and go to step 2.

Note that *w_i_* ^(*k*)^ represents the *i* ^th^ weighting coefficient at the *k* ^th^ iteration, **W** ^(*k*)^ is a diagonal matrix composed of *w_i_* ^(*k*)^, **x** ^(*k*)^ is the estimate of **x**, composed of *x_i_* ^(*k*)^, obtained from the *k* ^th^ iteration, and *ε* is a positive constant used to prevent *w_i_* ^(*k*+1)^ from becoming infinite. The empirical choice of *ε* is 0.1 to 1 and that of *k*_max_ is 4 to 8 [25]. It is also worth noting that the weighted *ℓ*_1_-minimization problem in step 2 is actually equivalent to
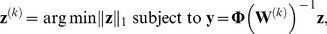
(17)so the reweighted *ℓ*_1_-minimization described above can be reduced to an *ℓ*_1_-minimization problem as expressed in (17) and thus simply solved by an *ℓ*_1_-minimization solver first. Then, we have **x** ^(*k*)^ = (**W** ^(*k*)^)^−1^ **z**. It should be also noted that here we adopted the Gradient Projection for Sparse Reconstruction (GPSR) algorithm [26] as the *ℓ*_1_-minimization solver, which has been already proved to be able to estimate HRV spectra in our previous study [24]. Furthermore, the IPFM-based spectral estimation problem as expressed in (9) can be exactly fit by the linear measurement model of CS as indicated in (13) simply by letting **Φ** = **A**.

## Results and Discussion

### Results on IPFM-synthesized Simulated HRV Signals

**Figure 1:**
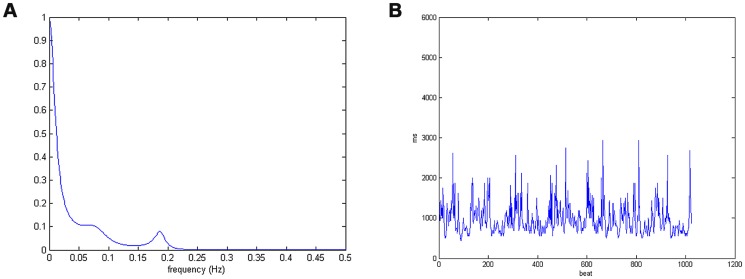
The theoretical AR model spectrum of the simulated modulating signal *m*(*n*) and its derived RR signal synthesized by inputting *m*(*n*) into the IPFM model: (a) the theoretical AR model spectrum, (b) an RR signal obtained from a realization.

In order to demonstrate the ability of reweighted *ℓ_1_*-minimization in estimating the IPFM-based HRV spectra under the CS framework, first we employed simulated HRV signals generated by the IPFM model. This is because real HRV signals are not adequate for performance evaluation since there is no way for one to know exactly what their actual spectra look like. Since a previous research in literature indicates that time-varying ARMA models may serve as a more realistic alternative for synthesizing the simulated HRV signals [27], in this study we analyzed spectra of HRV on AR derived simulated signals. Here, we simply used a similar AR model as suggested by [7] for simulating sequences of the modulating signal *m*(*t*). Denoting the discrete-time version of the modulating signal as *m*(*n*), we have employed the seventh order AR model for generating *m*(*n*), expressed as
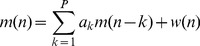
(18)where *a_k_*'s represent the model coefficients, *P* represents the model order (*P* = 7), and *w*(*n*) is the white noise with zero mean. All the numerical values of parameters related to this AR model are provided in Table 1. Here, the value used for sampling frequency is 1 Hz. This is because for HRV spectral analysis all the frequencies of interest generally fall below 0.5 Hz, thus setting the sampling frequency to 1 Hz is adequate. Fig. 1(a) shows the theoretical AR(7) model spectrum. According to Table 1, the seven poles are determined from the roots of the algebraic equation: 1–*a*_1_ *z* ^−1^−*a*_2_ *z* ^−2^−…−*a*_7_ *z* ^−7^ = 0, thus yielding the peak frequencies at 0 Hz, 0.0810 Hz, 0.1881 Hz, 0.3484 Hz, respectively.

**Table 1:** The numerical values of parameters related to the AR model used for generating the modulating signal *m*(*n*) when driven by zero-mean white noise *w*(*n*).

AR model parameters	Numerical values
Model order	*P* = 7
Model coefficients	*a*_1_ = 2.2683, *a*_2_ = −2.5629, *a*_3_ = 1.9455, *a*_4_ = −1.1488, *a*_5_ = 0.8494, *a*_6_ = −0.6892, *a*_7_ = 0.2923
Noise power of *w*(*n*)	*σ_w_* ^2^ = 0.005184
Signal power of *m*(*n*)	*σ_m_* ^2^ = 0.12937
AR poles	0.9303, 0.7306±*j*0.4075, 0.3540±*j*0.8645, −0.4157±*j*0.5845
Peak frequencies	0 Hz, 0.0810 Hz, 0.1881 Hz, 0.3484 Hz

The RR series were synthesized by inputting the simulated modulating signal *m*(*n*) into the IPFM model for obtaining the sequence of beat occurrence times. We generated 1024 heart beats in total for our numerical experiments. Fig. 1(b) shows an unevenly sampled RR signal corresponding to the AR model as indicated in (18). In order to evaluate the algorithm performance on the simulated RR signal from a comparative point of view, we here applied three methods for HRV spectral estimation as follows: the Lomb method, the *ℓ_1_*-minimization based CS method, and the proposed reweighted *ℓ_1_*-minimization based CS method. It should be noted that the Lomb power spectral estimation method was developed based on least-squares analysis [28]. It can be generalized to HRV spectral estimation on unevenly sampled data, thus avoiding the spectrum distortion due to the low-pass filtering effect resulting from the resampling process which is usually employed by classical FFT- or AR-based methods [7].

**Figure 2:**
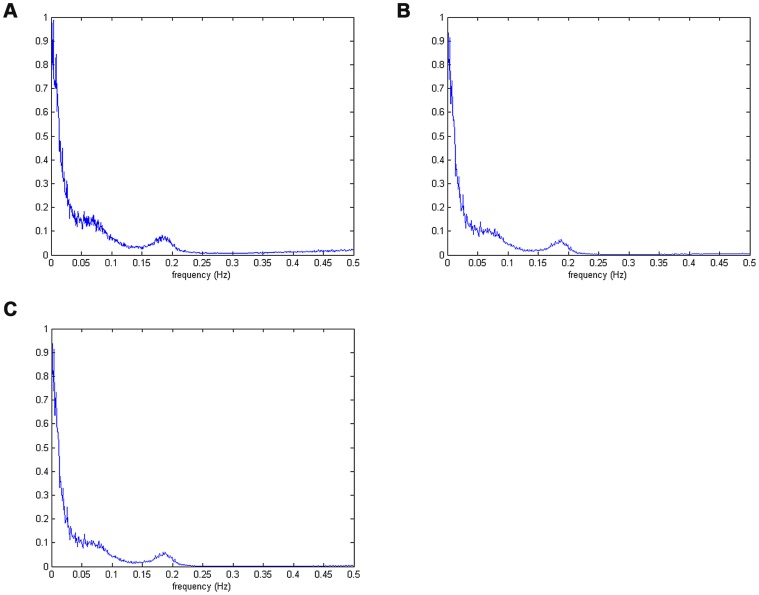
The mean spectra averaged over 100 realizations obtained from different methods: (a) Lomb, (b) *ℓ_1_*-minimization based CS, (c) reweighted *ℓ_1_*-minimization based CS.

Fig. 2 provides the mean spectra averaged over 100 realizations obtained from Lomb estimates, *ℓ_1_*-minimization based, and reweighted *ℓ_1_*-minimization based CS estimates, respectively. In fact, it is clearly revealed from Fig. 2 that while Lomb method introduces more high frequency contamination, both the *ℓ_1_*-minimization based and reweighted *ℓ_1_*-minimization based CS estimates can not only preserve the lower frequency components of HRV, but also attenuate the high frequency contamination. We then tested and evaluated these spectral estimates of unevenly sampled data using the Correlation Coefficient (CC). Given two vectors, denoted as **u** and **v**, respectively, the CC between them is calculated as
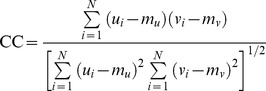
(19)where *u_i_* and *v_i_* are the elements of **u** and **v**, respectively; *m_u_* and *m_v_* are the mean values of *u_i_* and *v_i_*, respectively. Note that CC can be used to measure the similarity between the estimated spectrum and true spectrum. As a result, the performances, in terms of CC, obtained from Lomb, *ℓ_1_*-minimization and reweighted *ℓ_1_*-minimization were 0.9853, 0.9922 and 0.9922, respectively, suggesting that the CS framework, no matter based on *ℓ_1_*-minimization or reweighted *ℓ_1_*-minimization criteria, might achieve a better performance in spectral estimation than might the Lomb method.

In order to further demonstrate the power, in depth, of the reweighted *ℓ_1_*-minimization based CS in estimating HRV spectra from the incomplete set of RR measurements, we substantially shortened the simulated RR time series by removing a number of the RR intervals and then evaluated the algorithm performance by measuring the percent difference between the true spectrum and the spectrum derived from the incomplete simulated RR dataset. In this aspect, we experimentally studied the performance of spectral estimation on incomplete simulated HRV signals generated by the IPFM model to evaluate the robustness of the proposed reweighted *ℓ_1_*-minimization based CS method compared with other methods. First, a quantity used to represent the data loss rate, denoted as *R*, is defined as
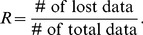
(20)


**Figure 3:**
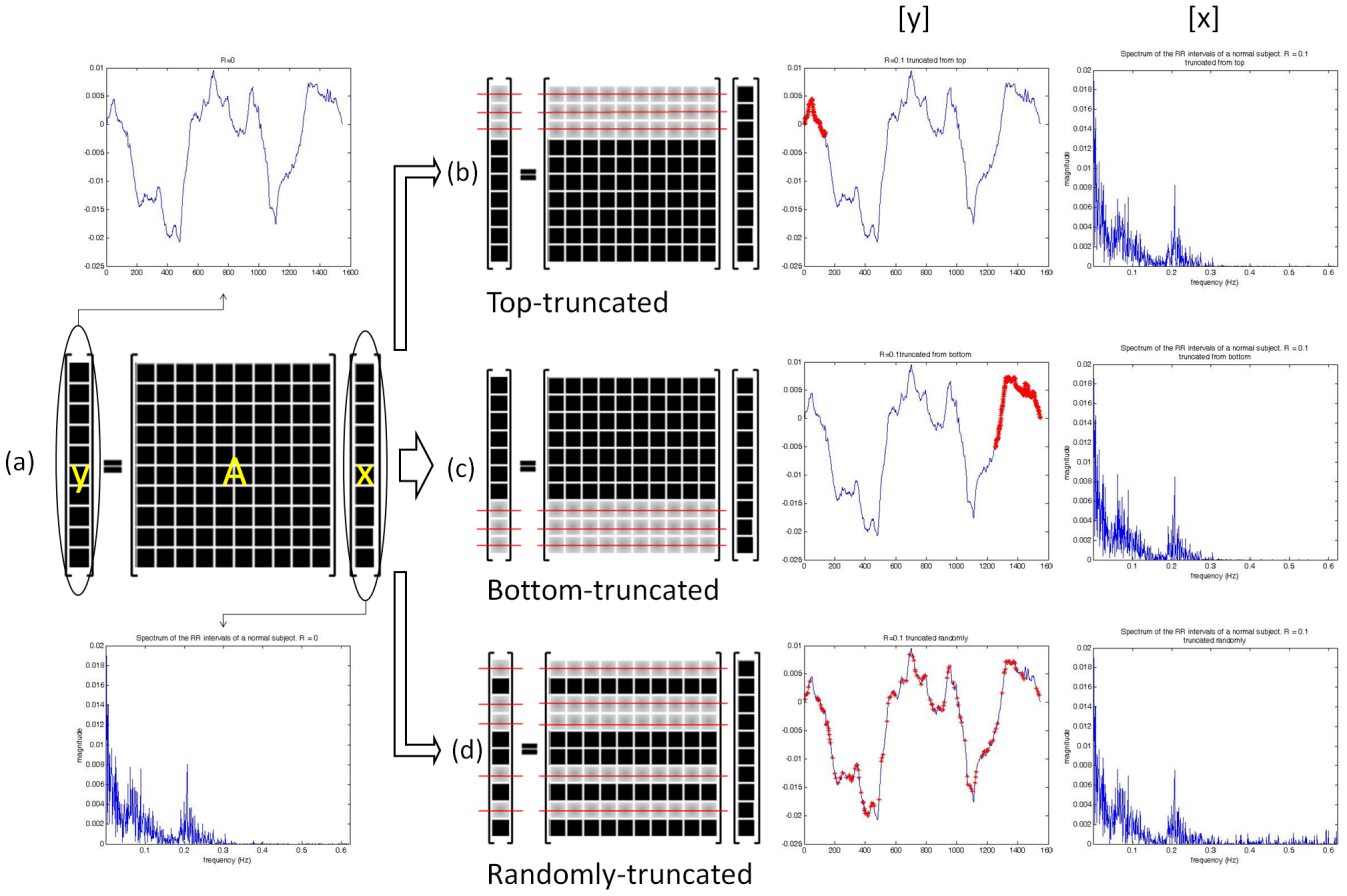
A schematic diagram describing how the proposed reweighted *ℓ_1_*-minimization based CS method is experimentally applied to HRV spectral estimation in the three data-truncation cases.

Our analysis scheme is devised as follows. Given an *R*, we removed a certain number of RR intervals accordingly in the top portion, in the bottom portion, and in a random order from the original simulated RR column vector to form incomplete RR datasets corresponding to the above three data-truncation cases, respectively. A schematic diagram, as shown in Fig. 3, is used to illustrate how the proposed reweighted *ℓ_1_*-minimization based CS procedure, as formulated in (9), is applied to HRV spectral estimation in the three data-truncation cases. Observing the diagram in Fig. 3, first note that in (a) **y** is a column vector formed by a given set of RR series and beat occurrence times as defined in (6); **x** denotes a sparse or compressible vector consisting of entries related to spectral estimates of the modulating signal and is as defined in (8). The HRV spectrum, in terms of **x**, is estimated using the reweighted *ℓ_1_*-minimization under CS framework. In the diagram, the upper leftmost plot gives an example of **y** obtained from a complete set of RR data and the bottom leftmost plot shows its corresponding spectrum derived from the CS estimates **x**. We then experimentally studied the performances of spectral estimation on incomplete RR datasets formed by removing a number of RR data in the top portion, in the bottom portion, and in a random order from the original RR data vector as illustrated in (b), (c), and (d), respectively.

With *R* gradually increased from 0 to 1 by a step size of 0.01, each time we estimated the HRV spectra from the incomplete RR datasets (i.e., for a given *R*) using the three different methods (*ℓ_1_*-minimization, reweighted *ℓ_1_*-minimization, and Lomb), respectively. To examine the capability of HRV spectral estimation from incomplete RR measurements, a quantitative measure, referred to as the Percent Error Power (PEP), was employed to evaluate the algorithm performance. It is defined as the percentage of the magnitude-squared difference between the theoretical spectrum (i.e., true spectrum) and the spectrum estimated from the incomplete simulated RR dataset with a specific data loss rate *R*, that is,
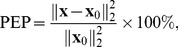
(21)where **x** and **x_0_** represents the estimated spectrum and the true spectrum, respectively.

**Figure 4:**
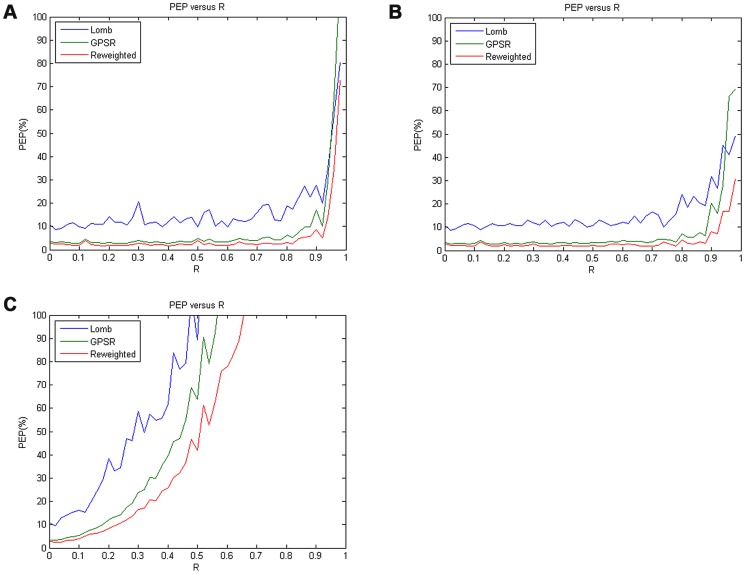
The PEP-versus-*R* curves derived from the three methods in each data truncated case: (a) the top-truncated case; (b) the bottom-truncated case; (c) the randomly-truncated case.

Fig. 4 presents the performance comparison in terms of PEP-versus-*R* curve among all the three methods in each data-truncation case as defined previously. Inspecting the plots in Figs. 4(a)–(c), we may see that in all the three cases, the reweighted *ℓ_1_*-minimization CS method apparently outperformed both the other two methods (i.e., Lomb and *ℓ_1_*-minimization CS methods) since its PEP estimates were always the smallest for any *R* values ranging from 0 to 1. In fact, we may see from all these plots that the PEP curves obtained from the reweighted *ℓ_1_*-minimization CS method always remained steadily the smallest throughout the entire *R* axis (ranging from 0 to 1), indicating that the proposed method could robustly achieve the best performance in HRV spectral estimation, compared with the other two methods, even under the situation of substantially fewer RR measurements. Moreover, it also appeared from Fig. 4 that in all the three data-truncation cases, the PEP estimates derived from Lomb estimation were always significantly larger than were those derived from the other two methods, respectively, under any given *R* values, thus showing the worst performance of all the three methods; one may also notice that while the performances of both the *ℓ_1_*-minimization and reweighted *ℓ_1_*-minimization CS methods were generally close to each other in all the three cases, the latter did outperform the former. On the other hand, it is worth noting that while all the PEP curves obtained from both the top- and the bottom-truncated cases (for all the three methods) steadily remained at certain levels until *R* = 0.75, those obtained from the randomly-truncated case all showed rapid, exponential-like growth as *R* increased; the PEP values obtained from the Lomb, *ℓ_1_*-minimization and reweighted *ℓ_1_*-minimization methods finally exceeded 100% at *R*≈0.5, 0.55, and 0.65, respectively.

Table 2 further provides the numerical results of averaged PEP produced by applying all the three methods to the AR-derived simulated HRV signals in each data-truncation case for *R*<0.2 (i.e., up to 20% data loss). It is revealed from the table that the proposed reweighted *ℓ_1_*-minimization based method always had the smallest PEP in all the three cases, indicating that the reweighted *ℓ_1_*-minimization actually achieved the best spectral estimation results (i.e., closest to the true spectrum) among all the three methods.

**Table 2:** The numerical results of averaged PEP produced by all the three methods for each data-truncation case (up to 20% data loss).

Method	Averaged PEP over *R* = 0∼0.2		
	Top-truncated	Bottom-truncated	Randomly-truncated
Lomb	10.7260%	10.5304%	18.7140%
*ℓ_1_*-minimization based CS	3.2279%	3.1482%	6.3028%
Reweighted *ℓ_1_*-minimization based CS	2.3373%	2.2712%	4.5521%

Furthermore, one may also see from Fig. 4 and Table 2 that no matter using what methods, the HRV spectral estimation performance in the random-truncation case was always degraded more significantly than that in both the top- and bottom-truncation ones. We may speculate that this should be due to the stationary nature of the AR-derived signals. Therefore, losing a significant amount of data in the top or bottom portion of the RR vector would not severely alter the characteristics of HRV spectrum. In contrast, the performance obtained from the randomly truncated RR dataset was continuously degraded when *R* was gradually increased, indicating that in the randomly-truncated case, the more the RR data points were lost, the more the major HRV spectral characteristics were destroyed.

**Figure 5:**
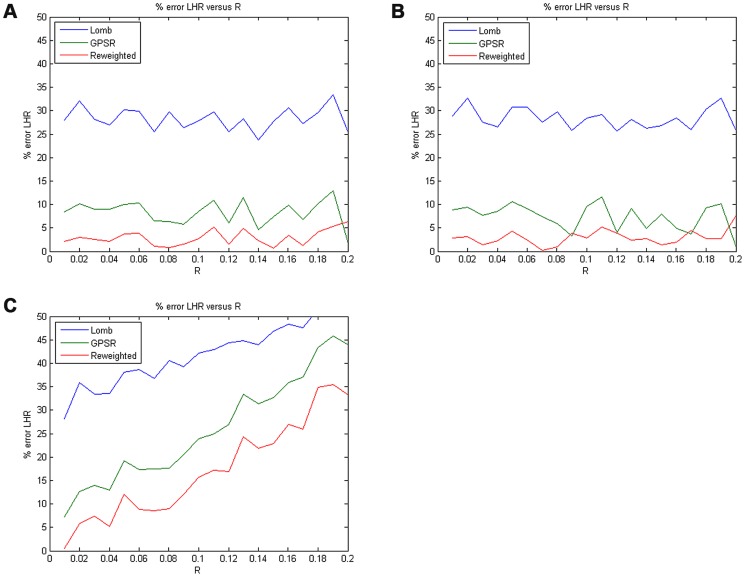
The %error-LHR-versus-*R* curves derived from the three methods in each data truncated case: (a) the top-truncated case; (b) the bottom-truncated case; (c) the randomly-truncated case.

In addition, since the HRV spectrum is normally divided into a Lower Frequency (LF) band [0.04,0.15] Hz and a Higher Frequency (HF) band [0.15,0.40] Hz, which correspond to the sympathetic and vagal activities, respectively [2], [3], in addition to using the PEP estimates the performance was also evaluated in terms of clinically relevant parameters, such as power in LF and HF bands, or in combination. Here, we have compared the relative spectral power ratio, LF/HF, obtained from each spectral estimation method with the theoretical value of LF/HF ratio derived from the original true HRV spectrum of *m*(*n*). In order to perform a quantitative performance evaluation, we defined the percent error LF/HF ratio (denoted as % error LHR) as
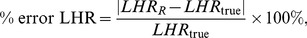
(22)where *LHR_R_* denotes the LF/HF ratio estimate derived from the incomplete sets of RR data with a specific data loss rate *R*; *LHR*_true_ represents the theoretical value of LF/HF ratio obtained from the original true HRV spectrum (*LHR*_true_ = 2.8693). Fig. 5 presents the performance comparison in terms of %error-LHR-versus-*R* curve among all the three methods in each data-truncation case as defined previously. Inspecting the plots in Figs. 5(a) and (b) first, we may see that in both top- and bottom-truncated cases, the reweighted *ℓ_1_*-minimization CS method apparently outperformed both the other two methods (i.e., Lomb and *ℓ_1_*-minimization CS methods) since its % error LHR estimates almost remained the smallest for any *R* values ranging from 0 to 0.2. This actually indicated that the LF/HF ratio estimates obtained from the reweighted *ℓ_1_*-minimization were generally closest to the theoretical value *LHR*_true_. On the other hand, one may also see that while the performances of both the *ℓ_1_*-minimization and reweighted *ℓ_1_*-minimization CS methods were close to each other, the latter still outperformed than the former.

Furthermore, it appeared from Fig. 5(c) that in randomly-truncated case, the %error-LHR-versus-*R* curves derived from all the three methods generally displayed a steady growth as *R* increased. Observing all the curves in Fig. 5(c), one may see that for a given *R* value, the reweighted *ℓ_1_*-minimization CS method always achieved the smallest % error LHR while the Lomb method always had the largest % error LHR.

On the other hand, in addition to the LF/HF ratio, we also calculated the VLF/HF ratio for assessing the proposed method in a similar way, as indicated in (22), since some medical studies consider VLF (0.0033∼0.04 Hz) as a relevant spectral region. Tables 3 and 4 provide the numerical results of averaged percent error in LF/HF ratio and in VLF/HF ratio, respectively, produced by applying all the three methods to the AR-derived simulated HRV signals in each data-truncation case (up to 10% and 20% data losses, respectively). It is revealed from both tables that no matter for up to 10% or 20% data loss, the proposed reweighted *ℓ_1_*-minimization based method always had the smallest percent error in both LF/HF and VLF/HF power ratio estimates in all the three cases, indicating that the reweighted *ℓ_1_*-minimization could substantially achieve the best HRV spectral fidelity among all the three methods. In addition, one may also notice that in the randomly-truncated case, both the averaged percent error in LF/HF and VLF/HF power ratio obtained from all the three methods were significantly increased as the *R* parameter was increased from 0.1 to 0.2 (for example, for the reweighted *ℓ_1_*-minimization method, the averaged percent error increased from 8.18% to 16.65% for LF/HF, and increased from 5.34% to 11.75% for VLF/HF), indicating the HRV spectral fidelity might start to be rapidly, severely degraded when *R* was at some values between 0.1 and 0.2.

**Table 3:** The numerical results of averaged percent error in LF/HF ratio (% error LHR) produced by all the three methods for each data-truncation case (up to 10% and 20% data losses, respectively).

Method	Averaged % error LHR over *R* = 0∼0.1		
	Top-truncated	Bottom-truncated	Randomly-truncated
Lomb	28.5885%	28.9245%	36.0012%
*ℓ_1_*-minimization based CS	8.6873%	8.3290%	15.8327%
Reweighted *ℓ_1_*-minimization based CS	2.5753%	2.6173%	8.1804%

Method	Averaged % error LHR over *R* = 0∼0.2		
	Top-truncated	Bottom-truncated	Randomly-truncated
Lomb	28.3798%	28.4378%	41.3909%
*ℓ_1_*-minimization based CS	8.4385%	7.5243%	25.2136%
Reweighted *ℓ_1_*-minimization based CS	3.0158%	3.0439%	16.6467%

**Table 4:** The numerical results of averaged percent error in VLF/HF ratio (% error VLHR) produced by all the three methods for each data-truncation case (up to 10% and 20% data losses, respectively).

Method	Averaged % error VLHR over *R* = 0∼0.1		
	Top-truncated	Bottom-truncated	Randomly-truncated
Lomb	40.2501%	39.9278%	47.1199%
*ℓ_1_*-minimization based CS	8.6467%	7.8905%	14.2949%
Reweighted *ℓ_1_*-minimization based CS	3.2365%	3.3187%	5.3391%

Method	Averaged % error VLHR over *R* = 0∼0.2		
	Top-truncated	Bottom-truncated	Randomly-truncated
Lomb	39.5630%	39.0032%	53.0711%
*ℓ_1_*-minimization based CS	6.5424%	6.0037%	22.2299%
Reweighted *ℓ_1_*-minimization based CS	4.2523%	5.1335%	11.7451%

As a result, all the numerical results as shown and described above actually indicated that the proposed reweighted *ℓ_1_*-minimization CS method could robustly achieve the best spectral fidelity in HRV assessment, compared with the other two methods, even under the situation of substantially fewer RR measurements. Since our study has shown that the reweighted *ℓ_1_*-minimization algorithm did achieve the best performance in HRV spectral estimation, we would further evaluate its performance on real HRV signals from a statistical point of view. The descriptions of the numerical experiments conducted on a real RR database of PhysioNet are presented in the subsequent subsection.

### Results on Real HRV Signals

In addition to evaluating the algorithm performance on simulated HRV signals from a comparative point of view as described above, in this study we also evaluated the performance of the reweighted *ℓ_1_*-minimization algorithm on a number of real HRV datasets from a statistical point of view. In order to achieve statistical significance, we here employed a set of real RR data from the interbeat (RR) interval databases of PhysioNet [29] for comprehensive examinations. In general, PhysioNet collects a large number of physiological signals and programs used to manipulate the signals. Almost all of the resources are free of charge. The testing database we adopted here is called the Normal Sinus Rhythm RR Interval (NSR-RRI) Database, comprising 54 datasets of RR intervals in total. In fact, the NSR-RRI database includes beat annotation files derived from 54 long-term ECG recordings of subjects in normal sinus rhythm (thirty men, aged 28.5 to 76, and twenty-four women, aged 58 to 73). The original ECG recordings (not available) were sampled and digitized at 128 samples per second, and the beat annotations were obtained by automated analysis with manual review and correction [30].

**Figure 6:**
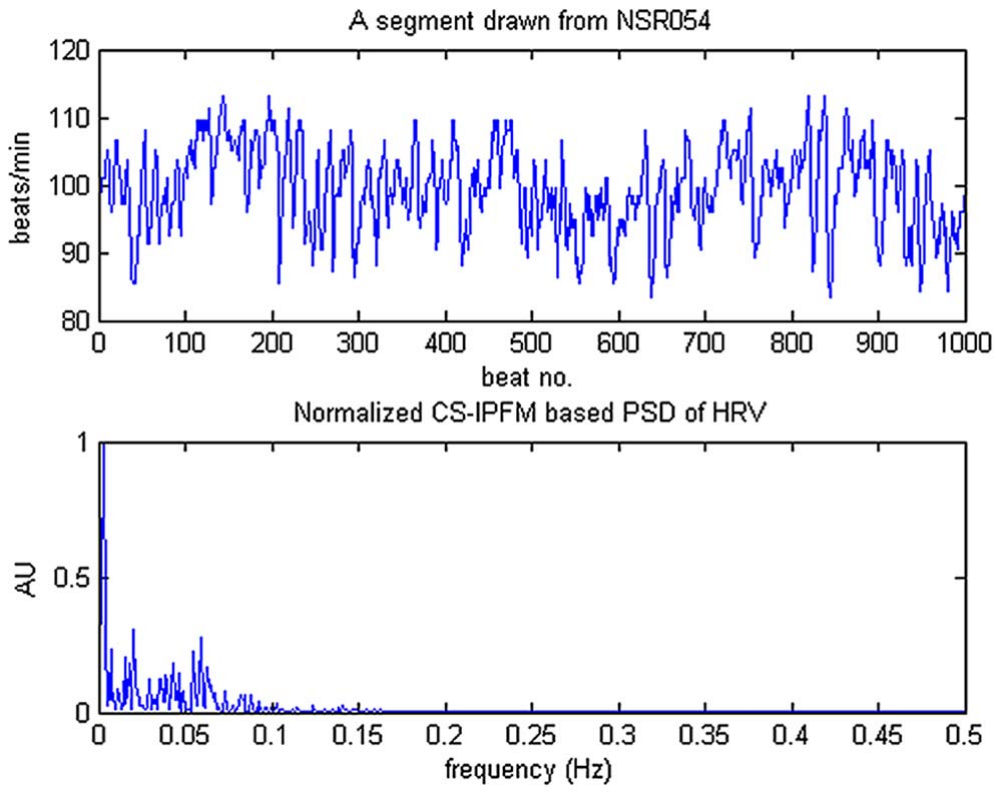
A 1000-point RR segment drawn from an NSR RR dataset (upper panel), and its corresponding normalized PSD derived from the reweighted *ℓ_1_*-minimization CS method (lower panel).

**Figure 7:**
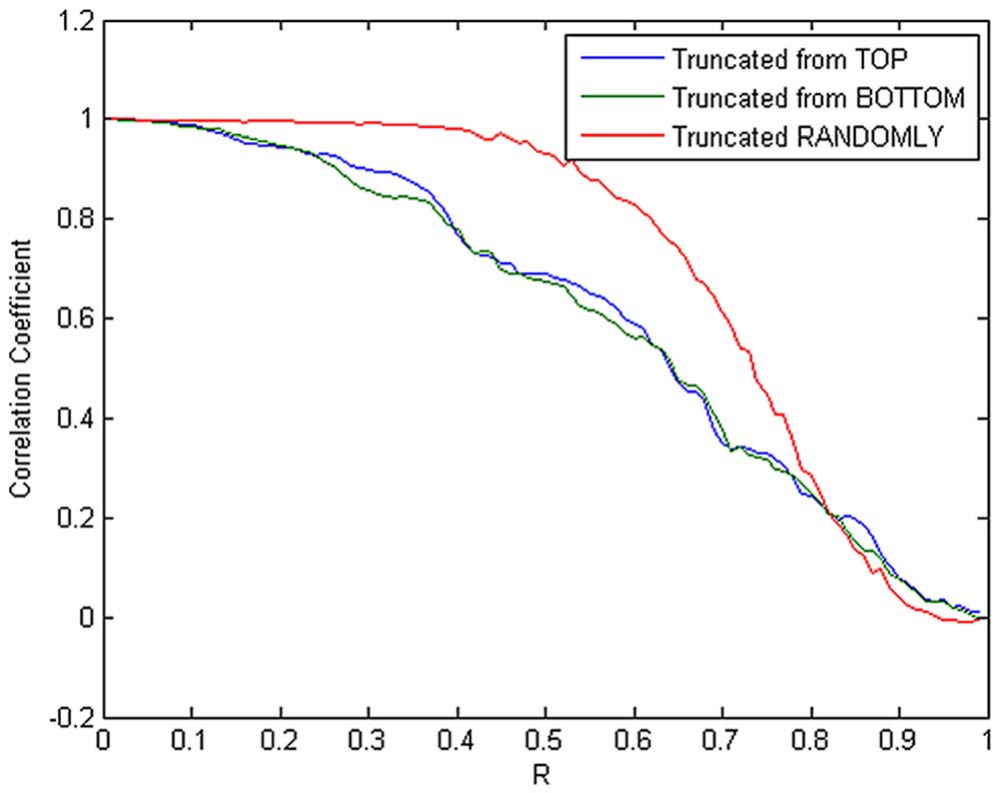
Mean CC-versus-*R* curve obtained by taking an average over the 54 realizations derived from the NSR-RRI Database of the PhysioNet in each data-truncation case.

Here, we took a 1000-point RR segment from each of the 54 RR datasets for our numerical experimental analysis. Fig. 6 shows a 1000-point RR segment drawn from an NSR-RRI dataset (NSR054) and its corresponding normalized PSD derived from the reweighted *ℓ_1_*-minimization CS method. Note that since there is no way for us to get the actual spectrum of a real HRV signal, we here simply used the spectral estimates derived from the original complete RR dataset as the true spectrum (i.e., without data loss or *R* = 0), and then evaluated the CC between the spectral estimates derived from the incomplete RR dataset with a given data loss rate *R*, ranging from 0.01 to 0.99, and the true spectrum. As a result, the mean CC-versus-*R* curve averaged over all the realizations derived from the 54 RR datasets of the NSR-RRI Database in each data-truncation case is calculated and illustrated in Fig. 7. It is indicated from the numerical results as shown in Fig. 7 that with CC remained above 0.95, the reweighted *ℓ_1_*-minimization based CS estimation not only can tolerate 17% and 19% data loss in the top-truncated case and the bottom-truncated case, respectively, but also can allow even a much higher data loss rate as 48% in the randomly-truncated case.

**Figure 8:**
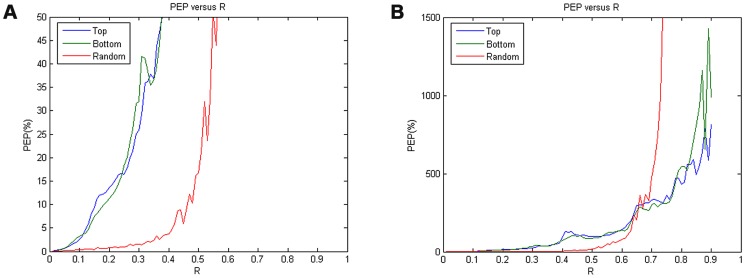
Mean PEP-versus-*R* curve obtained by taking an average over the 54 realizations derived from the NSR-RRI Database of the PhysioNet in each data-truncation case: (a) zoom-in version, (b) zoom-out version.

Moreover, it should be noted that since CC may not adequately reflect the small variance associated with a spectrum estimate, we further used the Mean Squared Error (MSE) based metric PEP, as indicated in (21), to evaluate the spectral estimation performance obtained from the reweighted *ℓ_1_*-minimization CS method in each data-truncation case. Fig. 8 shows the mean PEP-versus-*R* curve averaged over all the realizations derived from the 54 RR datasets of the NSR-RRI Database in each data-truncation case. Note that here we evaluated PEP by increasing *R* from 0 to 1. It is indicated from the numerical results as shown in Fig. 8(a) that with PEP remaining below 5%, the reweighted *ℓ_1_*-minimization based CS estimation not only can tolerate 12% and 13% data loss in the top-truncated case and the bottom-truncated case, respectively, but also can allow as even a much higher data loss rate as 41% in the randomly-truncated case. On the other hand, for up to 20% data loss the proposed method produced, on average, only 5.15%, 4.33%, and 0.39% PEP in the top, bottom, and random data-truncation cases, respectively.

Furthermore, one may see from Fig. 8(b) that the HRV spectral estimation performance in both the top- and bottom-truncation cases was always degraded more significantly than that in the random-truncation one when the data loss rate *R*<0.6. We may speculate that this should be due to the non-stationary nature of the real RR signals and thus, losing up to 10–20% of data in the top or bottom portion of the RR vector would severely alter the characteristics of HRV spectrum. In contrast, the performance obtained from the randomly truncated RR dataset appeared to be degraded much more quickly when *R* became large, say *R*>0.7, indicating that in the randomly-truncated case an incomplete RR dataset with over 70% loss in RR data would not be able to preserve the major HRV spectral characteristics anymore.

## Discussion

For online or mobile applications, it has been generally accepted that 5-minute ECG measurement is adequate for short-term HRV analysis [2], [31]. This choice of analysis time frame was actually imposed by the need to meet a good compromise between a sufficient frequency resolution and the signal stationarity which is required for reliable spectral estimation. However, such a choice may still be inadequately long. According to our numerical experimental results obtained from the performance evaluation, one may see the proposed reweighted *ℓ_1_*-minimization based CS method can still well recover most of the spectral estimates of HRV from a highly incomplete RR dataset formed by truncating a significant portion from the bottom of the original RR data vector. For example, inspecting the numerical results in terms of averaged percent error LF/HF ratio obtained by applying the proposed method to the bottom-truncated case as indicated in Table 3, we may see even up to 20% data loss would only result in about 3.04% averaged percent error in LF-to-HF power ratio estimate. Similarly, it is also indicated from Table 4 that for up to 20% data loss, the averaged percent error VLF/HF ratio was about only 5.13% in the bottom-truncated case for the proposed method. This actually implies for online short-term HRV analysis, while the conventional 5-minute measurement interval may be reduced up to 4 minutes so the short-time spectrum of HRV can be updated more quickly than before, about 95% of the spectral LF/HF or VLF/HF power ratio estimate would still remain unchanged with the same frequency resolution.

On the other hand, the missing or ectopic beats would cause abnormal beat-to-beat intervals, alternatively known as the outliers, which occur randomly and are longer or shorter than normal RR intervals. This actually represents a major source of error when analyzing HRV data in both the time and frequency domain. In particular, spectral estimation of HRV would be adversely affected by the presence of missing or ectopic beats, even only a small number of such beats. In fact, the potential impact of the proposed reweighted *ℓ_1_*-minimization algorithm is it can be robustly applied for either online or offline high-fidelity HRV spectral estimation, even under the situation of a certain degree of incompleteness in the RR data caused by ectopic or missing beats generated randomly. This has actually been implied by our numerical experimental results presented earlier in this paper. First, it is indicated from Tables 3 and 4 that up to 10% data loss in the randomly-truncated case would result in only about 8.18% and 5.34% averaged percent errors in LF-to-HF and VLF-to-HF power ratio estimates, respectively, suggesting that both the spectral LF/HF and VLF/HF power ratio estimates would still robustly remain at good estimation accuracy with the same frequency resolution. Note that such a performance is actually way better than good enough in actual practice since the typical outlier occupancy in an HRV signal should be within 5% in most cases. In addition, most of the existing QRS complex detectors may achieve even less than 1% false positives and false negatives (or above 99% detection accuracy) for the task of QRS detection [32].

About the limitations of the study, it should be noted first that in dealing with the situation involving the presence of outliers it is essential that an automatic detection of outliers in RR intervals be demanded before applying the proposed method to spectral analysis of HRV. In fact, the proposed method can be in conjunction with an existing RR outlier removal algorithm to estimate HRV spectra directly from RR intervals under the presence of outliers caused by ectopic beats. Although there are a number of RR outlier detection algorithms available [33], we are also developing such an algorithm that may facilitate the use of the proposed method in near future. Such an automatic detection mechanism may be also considered as a connection between the proposed method and its use for HRV assessment. In addition, it is also worth noting that the execution time of our method under MATLAB is generally within 1-2 seconds with 1000 data points. The processor type we used in generating all the results here was the X86 Family 6 Model 23 Stepping 10 GenuineIntel 3333 MHz CPU. Although it is suitable for online HRV analysis, it is still a little time consuming. In this aspect, since recent developments in Graphic Processing Unit (GPU) have shown its great potentials in accessing to high performance computing applications due to its massive multithreading capability, a GPU-based approach with the applications into the CS-IPFM based spectral analysis of HRV using a unified architecture, called Compute Unified Device Architecture (CUDA), can be further explored and evaluated in near future.

## Conclusion

In this study, a novel HRV spectral estimation method developed by combining the use of the IPFM model and the CS framework is proposed. The numerical results produced by tests conducted using AR model based simulated unevenly sampled RR data and a real RR database of PhysioNet both indicated that the reweighted *ℓ_1_*-minimization based CS technique was capable of achieving the best HRV spectral fidelity in comparison to the conventional *ℓ_1_*-minimization based CS and Lomb methods, even under the situation of substantially fewer measurements caused by either a reduction in RR data measurement time frame or the removal of RR outliers due to ectopic or missing beats.
